# Calmodulin Binding Proteins and Alzheimer’s Disease: Biomarkers, Regulatory Enzymes and Receptors That Are Regulated by Calmodulin

**DOI:** 10.3390/ijms21197344

**Published:** 2020-10-05

**Authors:** Danton H. O’Day

**Affiliations:** 1 Cell and Systems Biology, University of Toronto, Toronto, ON M5S 3G5, Canada; danton.oday@utoronto.ca; 2 Department of Biology, University of Toronto Mississauga, Mississauga, ON L5L 1C6, Canada

**Keywords:** calmodulin binding proteins, Alzheimer’s disease, neurodegeneration, glutamate receptors, calcium hypothesis, biomarkers, neurogranin, adenosine receptor, PMCA, ryanodine receptor

## Abstract

The integral role of calmodulin in the amyloid pathway and neurofibrillary tangle formation in Alzheimer’s disease was first established leading to the “Calmodulin Hypothesis”. Continued research has extended our insight into the central function of the small calcium sensor and effector calmodulin and its target proteins in a multitude of other events associated with the onset and progression of this devastating neurodegenerative disease. Calmodulin’s involvement in the contrasting roles of calcium/CaM-dependent kinase II (CaMKII) and calcineurin (CaN) in long term potentiation and depression, respectively, and memory impairment and neurodegeneration are updated. The functions of the proposed neuronal biomarker neurogranin, a calmodulin binding protein also involved in long term potentiation and depression, is detailed. In addition, new discoveries into calmodulin’s role in regulating glutamate receptors (mGluR, NMDAR) are overviewed. The interplay between calmodulin and amyloid beta in the regulation of PMCA and ryanodine receptors are prime examples of how the buildup of classic biomarkers can underly the signs and symptoms of Alzheimer’s. The role of calmodulin in the function of stromal interaction molecule 2 (STIM2) and adenosine A2A receptor, two other proteins linked to neurodegenerative events, is discussed. Prior to concluding, an analysis of how targeting calmodulin and its binding proteins are viable routes for Alzheimer’s therapy is presented. In total, calmodulin and its binding proteins are further revealed to be central to the onset and progression of Alzheimer’s disease.

## 1. Introduction

**Figure 1:**
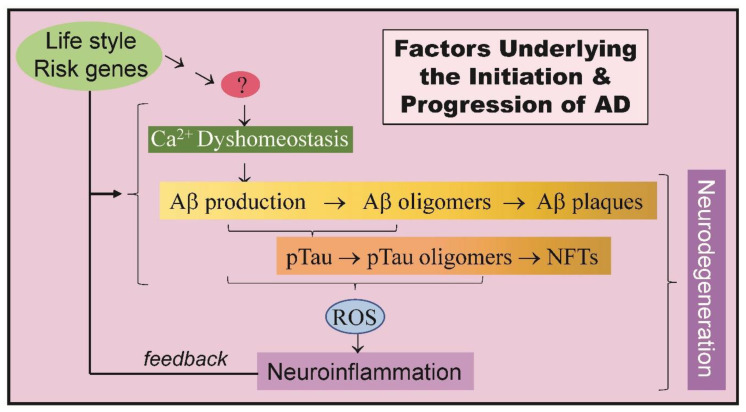
Factors underlying the initiation and progression of Alzheimer’s disease (AD). Lifestyle and Risk genes have a significant impact on the later development of AD but insight into how this occurs is not always clear. The initiating event(s) of AD (?) remain to be determined, but an early event is calcium dyshomeostasis that is implicated in the production of amyloid beta (Aβ) and phosphorylated Tau (pTau). Amyloid beta can form toxic oligomers that in turn can coalesce into amyloid plaques while pTau forms oligomers that form neurofibrillary tangles. These events are considered by many to lead to neurodegeneration. These, along with other events, generate reactive oxygen species (ROS) that underlies neuroinflammation an event in neurodegeneration but also one that can feedback to exacerbate or drive other events underlying AD.

For all diseases, finding the cause is paramount. With complex, multi-factorial diseases like Alzheimer’s disease (AD) finding ways to slow or stop its progression are of equal importance, especially when extremely large numbers of sufferers exist and those numbers continue to grow [1,2]. AD, the most prevalent cause of dementia, is characterized by amyloid beta (Aβ) plaques, neurofibrillary tangles and neuronal dysfunction which are widely believed to be underlie the neurodegenerative events associated with the disease. Despite a strong understanding of factors involved in AD, there is no available treatment that can significantly slow or stop its unrelenting progress (Figure 1). While traditional approaches to finding a “cure” have focused, and continue to focus, on the role of calcium dysregulation and preventing the accumulation of Aβ and neurofibrillary tangles, attention has also turned to events such as the accumulation of reactive oxygen species (ROS), cytokines and other bioactive molecules (e.g., [3]). The resultant neuroinflammation that can feed back to augment events linked to the disease, as well as contribute to the neurodegenerative process. Underlying all these processes and possibly driving them is the role of an individual’s lifestyle and the presence of mutations in a diversity of risk factor genes. Despite all of this information there is still a question about what actually causes AD. With the multiple points of view that exist and continue to grow, the search for a common element is getting swamped by an ever-increasing list of potential and possibly distracting targets. Here we show that calmodulin is involved in many of the central events associated with AD making it and its calmodulin binding proteins significant targets for future research.

Sixteen years ago, a central role for the small calcium-binding protein calmodulin (CaM) in AD was proposed [4]. This “Calmodulin Hypothesis” was a direct extension of the widely recognized and well-documented “Calcium Hypothesis”, which showed calcium dysregulation was a critical and early event in the development of AD [5,6]. While there is no doubt calcium is important, not only in normal neuronal function but in many aspects of neurodegeneration, it is but the tip of the functional iceberg that affects normal and Alzheimer’s brain cells. That is because calcium is not an effector of cell function but an ionic regulator that binds to and regulates downstream molecules, predominantly proteins. Calcium binding proteins do the work, not calcium. Thus, understanding the way that the dysregulation of calcium affects the onset and progression of AD requires an understanding of the proteins that calcium binds to and regulates. It can be argued that calcium has too widespread functions to serve as a target in the quest to stop the onset or progression of the disease. That said, the realization that calcium dysregulation is such a fundamental early event has set the stage for the next phase of research. Thus, it is critical to take all calcium-based studies one or more steps further to find specific downstream calcium-targets. As covered below, there are many reasons why CaM and its CaMBPs are those targets.

CaM is the major calcium binding protein of eukaryotic cells suggesting is a primary target of dysregulated calcium, in turn exerting its effects through its target CaM binding proteins (CaMBPs). The original theoretical data for the “Calmodulin Hypothesis” revealed that many the critical proteins in the amyloid processing pathway either were verified or potential CaMBPs that possess CaM-binding domains (CaMBDs) [4,7,8]. Subsequent research revealed that, as predicted, beta-secretase (BACE1; beta-site AβPP cleaving enzyme 1) and amyloid-β precursor protein (AβPP) both bind to and are regulated by CaM [9,10]. CaM binding to AβPP was verified by Canobbio et al. [11]. To add to the central role of CaM in amyloid processing ADAM10 (A Disintegrin And Metalloproteinase family member) the predominant α-secretase, binds CaM through an IQ-motif [12,13]. The multiple functions of CaM in the events of tau phosphorylation—through the regulation of calcium/CaM-dependent kinase II (CaMKII) and CDK5—and dephosphorylation (via CaN) have been analyzed [7,8]. Finally, the CaM binding attributes of many AD risk proteins was also dealt with in detail by [7,8].

Over the past 5 years, several publications have revealed that CaM’s role in AD extends much further and, as a result, continues to emphasize the potential significance of this small calcium binding protein as a target for therapeutic research, an approach already underway for the neurodegenerative Huntington’s disease. Here we update and review the most recent research supporting the link between CaM and Alzheimer’s focusing on a diversity of proteins linked to the onset and progression of the disease.

## 2. Biomarker Neurogranin and Synaptic Degeneration

Researchers continue to discover and evaluate potential biomarkers for AD. The key is to find combinations of biomarkers that define the various stages of the disease which in turn can assist not only in deciding potential patient treatments, but also in designing experiments and interpreting the results from clinical research. For example, two major groups of researchers, one from the Dominantly Inherited Alzheimer Network (DIAN) and the other from Alzheimer’s Disease Neuroimaging Initiative (ADNI), proposed four additional promising cerebrospinal fluid (CSF) biomarkers: chitinase-3-like protein 1 (YKL-40), neurogranin (Ng), synaptosomal-associated protein-25 (SNAP-25) and visinin-like protein 1 (VILIP-1) [14,15]. Of these Ng (also called RC3), a well-studied CaMBP, shows a lot of promise [16].

Synaptic loss is a critical event in the progression of AD preceding the neuronal loss associated with cognitive dysfunction [17,18,19]. As the most abundant post-synaptic CaMBP, Ng is an essential protein linked to the regulation of long-term potentiation (LTP) and long-term depression (LTD) [20,21]. LTP and LTD are forms of synaptic plasticity underlying the establishment and maintenance of various forms of memory. Localized to dendritic spines in associative cortical areas, Ng binds to calcium-free, apo-CaM via a full IQ motif (33IQASFRGHMARKKI46) thus restricting the availability of CaM for binding to other CaMBPs [22]. Ng functions, in part, to sequester CaM regulating local calcium/CaM signaling events [23,24,25]. Ng levels have been reported to be decreased in AD brains and increased in the CSF of individuals with mild cognitive impairment (MCI) [14,15,26,27,28,29]. Increased CSF levels of Ng are AD specific with high levels being indicative of rapid progression of the disease [28,30,31]. Ng levels are not only decreased in the AD brain, but the protein fails to localize at dendrites [32,33].

## 3. Neurogranin, LTP and LTD

CaM has critical but opposing functions in LTP and LTD. While many steps in the underlying events leading to LTP and LTD remain to be discovered various players have been revealed and critical proteins linked to each process have been identified. LTP induction requires a few second micromolar increase in local calcium levels coupled with the activation of NMDAR (NMDA receptor). Calcium binding to CaM allows it to activate CaMKII, which phosphorylates AMPAR (AMPA receptor), allowing the receptor to translocate to the synapse. AMPAR localization in the membrane provides evidence of LTP [20]. Conversely, a small increase in calcium results in CaM activating the sole CaM-dependent protein phosphatase calcineurin (CaN), which regulates LTD. Thus, depending on the intensity of short-term calcium fluxes within the same dendritic spine, CaM can either stimulate phosphorylation via CaMKII or initiate dephosphorylation through CaN leading to either LTP or LTD, respectively [21,34,35]. This opposing activation is partly due to the differential CaM-affinity of the two enzymes as well as to the spatial distribution of CaM and CaMKII in the spines along with Ng. Ng binds to apo-CaM, and thus has the potential ability to sequester, localize, concentrate and/or control the availability of this regulatory protein adjacent to the synaptic membrane [16,21,36].

## 4. CaMKII and Calcineurin Regulate LTP and LTD

O’Day et al. [8] reviewed the role of CaMKII and CaN, the two central CaM-dependent enzymes that play a cell signaling game of tug of war during AD, where they have opposite effects in dendritic spine maintenance and memory function. A recent review discusses how the localization and translocation of these two proteins in relation to NMDAR in post-synaptic densities in excitatory glutamatergic dendritic spines is critical to their opposing functions in the events of LTP (CaMKII) and LTD (CaN) [37]. These two signaling enzymes continue to reveal their significance in normal and disease function. During the last two years, several papers have extended our understanding of the importance of CaM in these events suggesting possible therapeutic routes for intervention.

As reviewed by Takemoto-Kimura et al. [38], CaM-dependent kinases (CaMKs: CaMKI, CaMKII, CaMKIV, CaMKK) have critical functions in cognition as a result of their importance in the development of neuronal circuitry, neuronal transmission, and synaptic plasticity. Most research has focused on the various forms of calcium-CaM dependent kinase II (CaMKII). Twelve subunits derived from four genes (α, β, δ, γ) form the CaMKII holoenzymes, each of which is activated by calcium/CaM binding [39]. Activation is followed by autophosphorylation (e.g., at T286 in αCaMKII) resulting in a stable, calcium independent CaMKII. As an example, the phosphorylation ability of activated p(T286)-αCaMKII is required for dendritic spine stabilization, LTP and memory formation. In contrast, CaN has been shown to destabilized spines, induce long term depression (LTD), and impair memory formation. As covered above, CaMKII is activated by high calcium levels, while lower levels activate CaN. While both CaMKII and CaN are dependent on calcium and CaM levels, how these levels affect the enzymes in microenvironmental locales remains to be determined.

Current research is focused on the Yin-Yang functions of these enzymes in AD: CaMKII the good and CaN the bad. The loss of p(T286)-αCaMKII activity at AD synapses is directly related to the severity of the disease [40]. Similarly, symptoms of the disease can be caused by the significant increase that occurs in CaN activity, likely caused by calpain-mediated cleavage of the protein as revealed by human AD brain extracts [41]. The take-home message from this work is that developing therapeutics that lead to increased CaMKII activity combined with approaches to decrease CaN activity appear to be a logical approach to dealing with some of the symptoms of AD. In support of this idea is evidence that transplant patients treated with the CaN inhibitor FK506 subsequently showed a reduced incidence of dementia [42]. This result is in keeping with research by others revealing that FK506 reduced Aβ burden and restored synaptic proteins and spine density in transgenic mice [43,44]. These results suggest that, regardless of the microenvironmental synaptic changes in calcium ions that occur during AD, the two prime targets of those changes (CaN, CaMKII) appear to be viable choices as therapeutic targets. Since they are both CaM-binding proteins, developing CaM-related peptides that bind to and enhance CaMKII coupled with such peptides that inhibit CaN are routes that could be taken.

The importance of CaMKII in AD was further revealed in a combinatorial study of drugs used to treat moderate to severe stages of the disease. Yabuki et al. [45] using memantine (uncompetitive N-methyl-D-aspartate receptor antagonist) and donepezil (cholinesterase inhibitor) together to treat olfactory bulbectomized mice lead to significant improvements in social interactions and depressive-like behaviors, as well as minor improvements in cognitive performance. These effects were linked in part to the decreased autophosphorylation of CaMKII.

## 5. Amyloid β Oligomers, mGluR and NMDARs

The importance of mGluR5 (metabotropic glutamate receptor 5), NMDAR (*N*-methyl-d-aspartate receptor) and AMPAR (α-amino-3-hydroxy-5-methyl-4-isoxazolepropionic acid receptor), three receptors that interact to regulate excitatory synaptic transmission, in AD has been reviewed [46]. During excitatory synaptic transmission, postsynaptic NMDARs and AMPARs respond differently to L-glutamate binding. AMPAR activation is fast, leading to millisecond membrane depolarizations while NMDAR activation is slower leading to the mobilization of downstream calcium-signaling pathways that underlie synaptic plasticity. As a result, these receptors have gained more attention in research on Alzheimer’s and other neurodegenerative diseases.

Increasing evidence argues for the importance of soluble Aβ oligomers as the toxic agents in AD pathogenesis (reviewed in [47]). The binding of CaM to Aβ monomers has been reviewed but the nature of CaM binding to oligomers has yet to be elucidated [8]. Aβ oligomers stop the induction of LTP by activating NMDARs containing GluN2B subunits (e.g., [48,49,50]. Opazo et al. [51] revealed that this occurs through the activation of CaMKII by Aβ oligomers, an event that has multiple downstream effects including stopping CaMKII autophosphorylation at T286, as well as the build-up and anchoring of synaptic AMPARs resulting in dendritic spine loss. Using cultured hippocampal neurons from E18 Sprague-Dawley rat embryos, they found that treatment with KN93, a CaMKII inhibitor, prevented both the LTP impairment and spine loss. However, while Opazo et al. [51] showed total CaMKII activity was activated by Aβ oligomers previous research had shown that Aβ oligomers inhibit CaMKII autophosphorylation [52,53]. The inability of Opazo et al. [51] to detect CaMKII autophosphorylation suggests more studies on this important area need to be undertaken. Regardless of the actual mechanism, the positive effect of using KN93, and potentially other CaMKII inhibitors and, possibly CaM antagonists, is supported but these results.

## 6. NMDARs Bind Calmodulin

There is still much more to be learned about the critical role CaM plays in regulating these events. The binding of CaM to the NR1 monomer of the heteromultimeric NMDAR and the role of this binding in allowing a negative feedback cycle was reviewed in detail by O’Day et al. [8]. CaM binds to the intracellular C0 and C1 domains of the NR1 subunit of tetrameric NMDARs enhancing the inactivation the receptor in response to an influx of calcium [54]. Inactivation leads to release of the receptor from the membrane. The C1 region contains a CaMBD with a 1–7 motif, a rare motif found also in MARCKs (myristoylated, alanine-rich, C-kinase substrate) [55]. The C1 region binds to other proteins (e.g., PKC) and is both necessary and sufficient for plasma membrane clustering but the role of CaM-binding to NMDAR in these events remains to be explored [56].

For example, the issue surrounding the regulation of NMDARs, and several other ion channels, is calcium-dependent inactivation (CDI). Recent research into this two-decade old issue, has added some insight. The binding of CaM to the intracellular C-term domain (C0) of the NMDAR NR1 subunit is mediates CDI [57]. Iacobucci and Popescu [58] showed that apoCaM was bound to NMDARs prior to their activation. This binding allows the receptor to be primed to sense calcium influx through the channel in response to ligand binding. Still, since CaMKII and other proteins are involved in NMDAR regulation, many aspects of this regulation remain to be elucidated.

CaM also binds to mGluR5 where it functions as a regulator of the trafficking of the receptor [59]. To add to this, CaMKIIα appears to mediate cross-talk between the two receptors where the kinase can bind mGluR5 until it is activated causing the enzyme to dissociate and then bind to an adjacent NMDAR GluN2B subunit [60]. Clearly, there is more to this interaction, but the take home message here is that CaM has multiple functions related to these two critical receptors in AD that in turn are affected by Aβ oligomers. The CaM story does not stop there, however.

## 7. LTP and LTD Regulation by Calmodulin

Thus, there is a critical interplay between Ng, CaM, CaMKII and CaN in LTP and LTP, two events in synaptic plasticity [21,25]. A few second, large increase in local post-synaptic calcium ion concentration leads to a conformational change in CaM in turn permitting it to bind to and activate CaMKII a critical step in LTP. In contrast, a small increase fails to lead to CaMKII activation but instead allows CaM to activate CaN, an essential step in LTD. Since the amounts of CaMKII, CaN and other CaMBPs greatly exceed the amount of CaM available, CaM is the limiting factor in regulating these events. CaM availability is also limited by post-synaptic CaMBPs called calpacitins that bind apo-CaM limiting CaMs ability to activate CaMBPs [36,61]. Ng is a calpacitin that has been shown to target CaM to the post-synaptic membrane and, by localizing it, enhancing sensitivity to calcium ion levels in turn fine-tuning the regulation of CaMKII and CaN [25,62].

**Figure 2:**
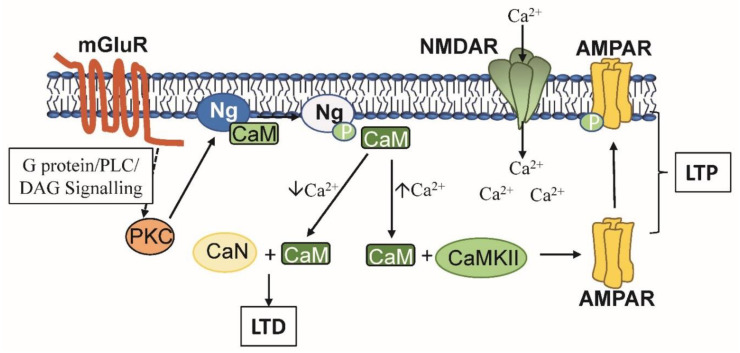
A model for LTP involving regulation mediated by calmodulin (CaM) and neurogranin (Ng). Activation of NMDARs causes a calcium influx activating Ca^2+^/CaM that activates CaMKII. CaMKII phosphorylates AMPARs leading to their translocation and insertion into the post-synaptic membrane. Activation of mGluR initiates G protein/PLC/DAG mediated signaling to activate PKC. PKC phosphorylates Ng preventing CaM binding and allowing CaM to continue activating CaMKII. See text for details.

The above information on the role of CaM in regulating LTP and LTD is summarized in Figure 2. Activation of NMDARs in the post-synaptic membrane leads to a large calcium influx activating calcium/CaM which in turn binds to and activates CaMKII. CaMKII phosphorylates AMPARs leading to their translocation and insertion into the synapses, evidence of LTP [63]. Ligand binding to mGluR initiates G protein/PLC/DAG mediated signaling that activates PKC. PKC phosphorylates Ng removing its ability to bind to CaM. This allows CaM to remain free to activate CaMKII longer, a potentially essential event for LTP [63].

## 8. CaM and Amyloid Beta Regulate PMCA

**Figure 3:**
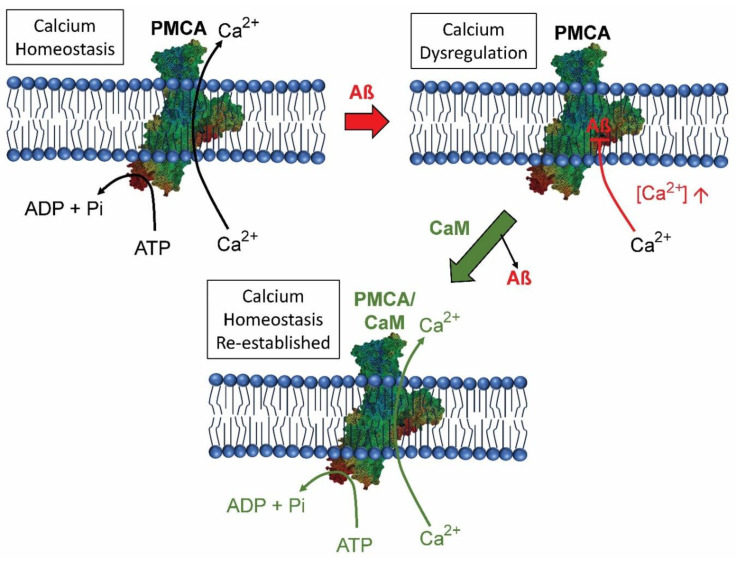
The regulation of PMCA and calcium levels by amyloid beta (Aβ) and calmodulin (CaM). The calcium-pump PMCA is critical to calcium homeostasis. In the presence of Aβ, PMCA binds to and is inhibited by the neurotoxic peptide, leading to a buildup of intracellular calcium. CaM does not only bind to Aβ, it also significantly increases the calcium pump activity of PMCA. While describing each of these proven events, this scenario suggests that CaM could play a therapeutic function in re-establishing calcium-homeostasis caused by Aβ.

O’Day et al. [8] reviewed the work of Berrocal et al. [64], who revealed a protective role for CaM in controlling calcium dysregulation, a central event in AD. Plasma membrane calcium ATPases (PMCAs) are CaM-binding ion pumps found in the cell membrane of all eukaryotic cells [65]. They function to maintain calcium ion homeostasis by eliminating the ions from cells. The CaM-binding autoinhibitory C-term tail of the protein keeps the pump inactive at low calcium levels. PMCAs are also the only brain calcium-pumps that bind to and are inhibited by Aβ. CaM is the primary cellular activator of PMCA (Figure 3). When CaM binds to PMCA, Aβ is no longer free to inhibit PMCA allowing calcium entry into cells already subjected to calcium dysregulation. More recent work by Corbacho et al. [66] not only validated and clarified the calcium-dependent binding of CaM to Aβ, but also revealed that CaM binds with high affinity to its neurotoxic domain (Aβ25-35) leading to the inhibition of Aβ fibril formation. Based on their results, the authors suggested CaM as a primary therapeutic target, as previously proposed [7,8].

However, the PMCA regulatory story does not stop there. Tau that is present in membrane vesicles can bind to and inhibit the activity of PMCA [67]. CaM, the major PMCA activator, prevents this inhibition at nanomolar concentrations. The tau binding site is in the C-term tail of PMCA close to the CaM-binding domain but how this competitive interaction works is under analysis.

## 9. Ryanodine Receptors, CaM and AD

Recent data suggests ryanodine receptors (RyRs) could be favorable therapeutic targets for treating AD and other disorders [68]. The endoplasmic reticulum-based, calcium-channel, RyRs have been revealed to be important functionaries in the calcium dysregulation linked to AD, where they have a proposed dual function [69]. Overexpression of AβPP (APP_695_ or APPS_WE_) in Tg2576 (APPS_WE_) mice results in increased RyR expression and enhanced calcium release from the RyR channels [70]. Reduction of RyR-mediated calcium release with dantrolene led to decreased Aβ levels and Aβ-associated histological lesions with an associated decrease in learning and memory defects.

**Figure 4:**
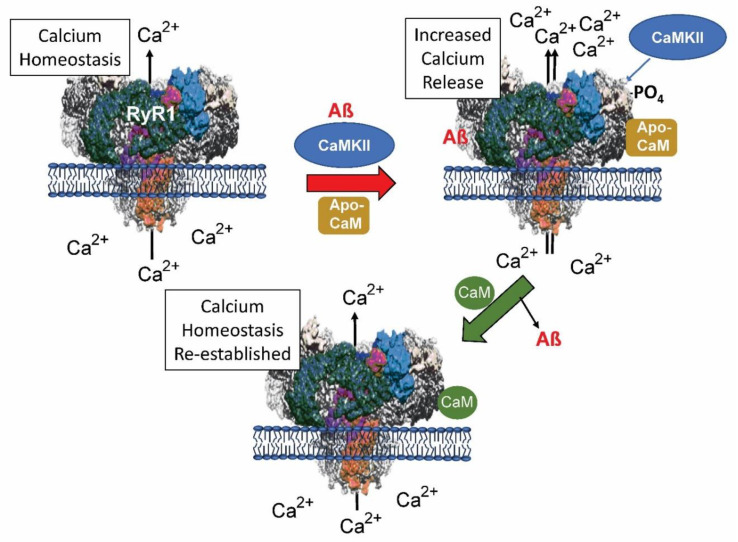
The effects of calmodulin (CaM) and amyloid beta (Aβ) on calcium release via RyR1 from the endoplasmic reticulum. Binding of Aβ or phosphorylation of RyR1 by the calcium/CaM-dependent kinase II (CaMKII) can lead to increase calcium release from the endoplasmic. Binding to calcium-free apo-CaM can also generate higher levels of calcium release. Calcium-bound CaM slows calcium release in normal cells and as projected here, could possibly restore dysregulated levels to normal.

All three RyR channels (RyR1-3) bind CaM (one CaM/subunit of the RyR tetramer; reviewed in [71,72]). In the early stages of AD, the RyRs compensate for the calcium disruption while in later stages they contribute to the increasing dysregulation. Part of this is due to the occurrence of calcium-induced calcium release (CICR) from the RyRs, which is caused by high influxes of calcium across the cell membrane [73]. The multiple reasons for this are under analysis but evidence implicates both Aβ and APP as relevant factors. For example, Aβ production is increased as a result of the release of calcium from RyR and in turn Aβ increases calcium release from RyR channels [74,75]. Research into the effects of CaM binding on RyR and calcium release is limited. However, it has been shown that apo-CaM is an agonist for RyR1 while calcium/CaM inhibits the receptor and the release of calcium ions [72]. CaMKII regulates RyR1 by phosphorylating specific residues leading to an increase in calcium release [76]. These data are summarized and extended in Figure 4.

## 10. Orai and STIM2: A Calcium Sensor and Calmodulin Binding Protein

Another protein linked to AD has entered the calcium/CaM/AD realm. STIM2 (stromal interaction molecule 2) is a multifunctional protein originally identified as a resident of the ER, where it is involved in regulating the levels of calcium in the ER and cytoplasm via store-operated calcium entry (SOCE) [77,78,79]. STIM2 has been shown to be involved in AD, autoimmune disorders, cancer and Huntington’s disease. As detailed in a review by Berna-Erro et al. [77], SOCE involves a complex interaction between the CRAC complex (calcium release-activated calcium channels), STIM1 (Stromal interaction molecule 1), Orai1 (Calcium Release-Activated Calcium Modulator 1), TRPC1 (transient receptor potential canonical 1 and other proteins) [80]. These proteins define the function of STIM2 in regulating calcium levels, a function regulated by CaM. Two of the three STIM2 isoforms (STIM2.1, STIM2.2), generated by alternative splicing, have a CaM-binding domain in their C-term, the region of interaction with STIM1, CRAC, Orai and TRPC. The CaM and Orai binding sites overlap and are mutually exclusive suggesting a critical calcium-dependent regulatory interaction. STIM2 colocalizes with CaMKII in hippocampal mushroom (mature) spines thus linking it to long term potentiation and postsynaptic plasticity [77,81]. In keeping with this, among other things, STIM2-deficient mice show extensive neuronal loss in their hippocampus [81]. More direct results have implicated STIM2 in AD. STIM2 expression and SOCE were reduced in murine embryonic cells expressing mutated presenilins, and this downregulation of STIM2 was associated with the loss of mushroom spines. What is more, STIM2 overexpression in two AD mouse models corrected the phenotypes [81]. This small overview of STIM2 reveals its central role in calcium homeostasis, its functional regulation by CaM, as well as links to AD, thus adding one more level of relevance to the Calmodulin Hypothesis.

The role of CaM in SOCE and its link to STIM2 function and AD does not end there. STIM1 also binds to CaM, an event that disrupts its binding to Orai1 leading to the closing of the Orai1 calcium channel [82]. The role of TRPC channels as therapeutic targets in AD has been reviewed, and several TRPC isoforms have been shown to be regulated by CaM [83,84].

## 11. Adenosine A2A Receptor

The purine ribonucleoside adenosine is recognized for its neuromodulating abilities including the regulation of Alzheimer’s-related cognitive function [85]. There are four G-protein coupled adenosine receptors: A1, A2A, A2B, A3. A1 and A2A are involved in learning and memory. The adenosine receptor antagonist caffeine, which acts through A1 and A2A, has been shown to improve cognitive function in AD sufferers and reduce Aβ load and tau burden while improving cognition in transgenic animal models [86]. The positive results have been replicated with other antagonists of adenosine A2A receptors. Co-treatment of neuronal cell cultures with a diversity of other receptor antagonists plus Aβ led to the complete prevention of Aβ-induced neurotoxicity [85].

Both A1 and A2 receptors have been implicated in the pathogenesis of AD. For example, knocking out A2AR (adenosine A2A receptor) or blocking its function pharmacologically in a tau-transgenic mouse model protected against tau-induced deficits in spatial memory and long-term depression [87]. It has been proposed that symptomatic relief from cognitive impairment in AD sufferers could result from the modulation of A2AR. A2AR binds CaM intracellularly in the C-terminus of the protein adjacent to the cell membrane [88]. The arginine-rich CaM-binding sequence (291RIREFRQTFR300) has been defined [89]. A Calmodulin Target Database scan of human adenosine receptor A2a (P29274) revealed this sequence fell within an extended CaM binding domain (FIYAYRIREFRQTFRKIIRSH) within which several binding motifs are present (i.e., 1-10, two 1-14, 1-12 and 1-16), indicating that the arginine rich region may allow binding to CaM in various ways. The question now is how this domain impacts A2AR function and its potential use as a therapeutic in AD and other neurodegenerative diseases.

## 12. Targeting Calmodulin in Dementia

As detailed above, the evidence is compelling that CaM and its CaMBPs function in multiple critical events at all stages of both early and late onset AD. The question now is how to use this information to prevent, slow or stop the progression of the disease. The answer is that therapies using CaM antagonists and inhibitors of the CaM-dependent enzymes CaMKII and CaN have already been employed for several decades for the treatment of other conditions. Recently there has been a renewed enthusiasm for their use in treating various diseases including neurodegeneration.

Target-specific antagonists already exist for the CaM-regulated enzymes CaMKII and CaN [8,44]. For example, the CaN inhibitor FK506 has been shown to be effective at reversing object recognition defects in Tg2576 mice. Tg2576 is a well characterized mouse models of AD that overexpresses the Swedish APP mutation (KM670/671NL) leading to elevated levels of Aβ and amyloid plaques [90]. Dendritic spine, and neuron morphology improved in YFP-APP/PS1 transgenic mice treated with this inhibitor. What is more is that the inhibition of CaN post-surgery in human after solid organ transplants led to a lower incidence of dementia [42]. The treatment of a Huntington’s mouse model (R6/2) with a peptide derived from the CaM sequence resulted in neuroprotection likely through the inhibition of CaM binding to the huntingtin protein [91,92]. More recently, [93,94] used polysialic acid-based micelles to effectively cross the blood-brain barrier (BBB) to deliver a CaM antagonist (DY-9836) for the treatment of vascular dementia. Their CaM inhibition research on the treatment of vascular dementia and bilateral carotid artery stenosis led to cognitive improvements possibly via the inhibition of nitric oxide over production (i.e., nitrosative stress) and inflammasome activation events involving the CaMBPs CaN and CaMKII. These studies and others reported above support the concept of targeting CaM and its binding proteins in the Alzheimer’s brain and provide mechanisms for doing so.

Other CaM targeting pharmaceuticals have also been proven to be safe for use in people. CaM antagonists have recently been successfully used to treat a diversity of cancers including pancreatic cancer and cancer-dependent events such as angiogenesis [95,96]. The development of several novel CaM and CaMBP antagonists/inhibitors with therapeutic value adds to the already long list from which researchers into Alzheimer’s and other neurodegenerative diseases can use immediately [95,97].

## 13. Conclusions

The EF hand is a calcium ion-binding helix–loop–helix motif present in calcium-binding proteins. There is no doubt that CaM has its EF hands in a diversity of essential life functions, so it comes as no surprise that it is also be linked to critical events in AD. As a central player coupled with the validated importance of calcium dysregulation, it is disconcerting that more has not been done on the subject. Targeting CaM with CaM-peptides has already proven to be of use in treating Huntington’s disease patients while inhibiting the calcium-CaM-dependent protein phosphatase CaN reduces the incidence of dementia [7,8,42,44,90,91]. With the large number of CaM antagonists that already exist, as well as insight into the CaM-binding domains of multiple proteins linked to AD, it would seem that clinical trials in this area will have a head start.

Other than the classic hallmarks of AD—Aβ, Aβ oligomers, Tau/pTau—CaM is the only protein that is linked to essentially all the central pathways involved in the disease (Table 1). CaM and its binding proteins are involved in multiple events in the amyloid pathway and neurofibrillary tangle formation [4,7,8]. Many of the major risk factor proteins identified by GWAS have either been proven to bind to CaM or possess CaMBDs. CaM has a central role in learning, memory and synaptogenesis. Critical calcium ion channel receptors (mGluR, NMDAR, RyR1), proteins linked to calcium regulation (Orai/STIM2) and other receptors (A2AR) are CaMBPs. Thus, its potential as a therapeutic target appears to surpass any other protein. The key now is to employ proven and effective pharmaceuticals to target CaM and/or specific CaMBPs possibly in combination with other AD-based pharmaceuticals.

## Figures and Tables

**Table 1:** Verified calmodulin binding proteins directly linked to Alzheimer’s disease.

Protein	Function in AD
**Amyloid Pathway**	
Amyloid β (Aβ) ^1,2^	Main component of amyloid plaques
Amyloid β precursor protein 1 (AβPP1) ^1,2^	Source of Aβ
β-Secretase 1 (BACE1) ^1^	1st enzyme in amyloid pathway
Presenilin-1 (PSEN-1) ^1,2^	Component of γ-secretase
**Neurofibrillary Tangle Formation**	
Tau (MAPT) ^1^	Microtubule binding, neurofibrillary tangles
Calcium/calmodulin dependent protein kinase II (CaMKII) ^1,2^	Tau phosphorylation; memory, etc.
Calcineurin (Protein phosphatase 2b) ^1,2^	Tau phosphorylation; memory, etc.
**Critical Receptors and Ion Channels**	
Adenosine receptor A2 (AdoA2) ^2^	Inhibition improves cognitive function
Metabotropic muscarinic receptors (mAchR) ^1^	Cholinergic hypothesis; neurocommunication
N-methyl-D-aspartate receptor (NMDAR) ^1^	Synaptic plasticity; memory
plasma membrane Ca^2+^-ATPase (PMCA) ^1,2^	Calcium homeostasis
Ryanodine receptors (RyR1-3) ^2^	Calcium homeostasis
Store Operated Calcium Entry (Orai/STIM2) ^2^	Calcium homeostasis
**Risk Factors and Microglia**	
ATP-binding cassette transporter A7 (ABCA7) ^1^	Transport of Aβ across BBB to blood
Bridging integrator 1 (BIN1) ^1^	AßPP1 endocytosis; susceptibility gene
**Synapse and Neuron Loss**	
Neurogranin ^2^	LTP, memory, cognition

^1^ Reviewed and references in O’Day et al. [8] and O’Day [7]; ^2^ Discussed and references in this article.

## Abbreviations

A2AR	Adenosine A2A receptor
Aβ	Amyloid beta
AβPP	Amyloid-β precursor protein
AD	Alzheimer’s disease
AMPAR	α-Amino-3-hydroxy-5-methyl-4-isoxazolepropionic acid receptor
BACE1	Beta-secretase 1
CaM	Calmodulin
CaMBD	CaM-binding domain
CaMBPs	CaM binding proteins
CaMKII	Calcium/CaM-dependent kinase II
CaN	Calcineurin
CRAC	Calcium release-activated calcium channels
CSF	Cerebrospinal fluid
*EF hand*	Calcium ion-binding helix-loop-helix motif
LTP	Long-term potentiation
LTD	Long-term depression
MARCKs	Myristoylated, alanine-rich, C-kinase substrate
mGluR5	Metabotropic glutamate receptor 5
NFTs	Neurofibrillary tangles
Ng	Neurogranin
NMDAR	*N*-methyl-d-aspartate receptor
Orai1	Calcium Release-Activated Calcium Modulator 1
PMCA	Plasma membrane calcium ATPase
pTau	Phosphorylated Tau
ROS	Reactive oxygen species
RyRs	Ryanodine receptors
STIM1, 2	Stromal interaction molecule 1, 2
TRPC1	Transient receptor potential canonical 1

## References

[1] (2020). Alzheimer’s Association Report: 2020 Alzheimer’s disease facts and figures. Alzheimers Dement..

[2] Livingston G., Huntley J., Sommerlad A., Ames D., Ballard C., Banerjee S., Brayne C., Burns A., Cohen-Mansfield J., Cooper C. (2020). Dementia prevention, intervention, and care: 2020 report of the Lancet Commission. Lancet.

[3] Tanaka M., Toldi J., Vécsei L. (2020). Exploring the Etiological Links behind Neurodegenerative Diseases: Inflammatory Cytokines and Bioactive Kynurenines. J. Mol. Sci..

[4] O’Day D.H., Myre M.A. (2004). Calmodulin-binding domains in Alzheimer’s disease proteins: Extending the calcium hypothesis. Biochem. Biophys. Res. Commun..

[5] Khachaturian Z.S. (1994). Calcium hypothesis of Alzheimer’s disease and brain aging. Ann. N. Y. Acad. Sci..

[6] Marx J. (2007). Fresh evidence points to an old suspect: Calcium. Science.

[7] O’Day D.H. (2019). Alzheimer’s Disease: A short introduction to the calmodulin hypothesis. AIMS Neurosci..

[8] O’Day D.H., Eshak K., Myre M.A. (2015). Calmodulin Binding Proteins and Alzheimer’s Disease: A Review. J. Alzheimers Dis..

[9] Chavez S.E., O’Day D.H. (2007). Calmodulin binds to and regulates the activity of beta-secretase (BACE1). Current Research on Alzheimers Disease.

[10] Myre M.A., Tesco G., Tanzi R.E., Wasco W. Calmodulin binding to APP and the APLPs. In Molecular Mechanisms of Neurodegeneration. Proceedings of the A Joint Biochemical Society/Neuroscience Ireland Focused Meeting.

[11] Canobbio I., Catricalà S., Balduini C., Torti M. (2011). Calmodulin regulates the non-amyloidogenic metabolism of amyloid precursor protein in platelets. Biochim. Biophys. Acta..

[12] Nagano O., Murakami D., Hartmann D., De Strooper B., Saftig P., Iwatsubo T., Nakajima M., Shinohara M., Saya H. (2004). Cell-matrix interaction via CD44 is independently regulated by different metalloproteinases activated in response to extracellular Ca^2+^ influx and PKC activation. J. Cell Biol..

[13] Kuhn P.H., Wang H., Dislich B., Colombo A., Zeitschel U., Ellwart J.W., Kremmer E., Rossner S., Lichtenthaler S.F. (2010). ADAM10 is the physiologically relevant, constitutive alpha-secretase of the amyloid precursor protein in primary neurons. EMBO J..

[14] Schindler S.E., Li Y., Todd K.W., Herries E.M., Henson R.L., Gray J.D., Wang G., Graham D.L., Shaw L.M., Trojanowski J.Q. (2019). Emerging cerebrospinal fluid biomarkers in autosomal dominant Alzheimer’s disease. Alzheimers Dement..

[15] Sutphen C.L., McCue L., Herries E.M., Xiong C., Ladenson J.H., Holtzman D.M., Fagan A.M. (2018). On behalf of ADNI, 2018. Longitudinal decreases in multiple cerebrospinal fluid biomarkers of neuronal injury in symptomatic late onset Alzheimer’s disease. Alzheimers Dement..

[16] Liu W., Lin H., He X., Chen L., Dai Y., Jai W., Xue X., Tao J., Chen L. (2020). Neurogranin as a cognitive biomarker in cerebrospinal fluid and blood exosomes for Alzheimer’s disease and mild cognitive impairment. Translat. Psych..

[17] DeKosky S.T., Scheff S.W. (1990). Synapse loss in frontal cortex biopsies in Alzheimer’s disease: Correlation with cognitive severity. Ann. Neurol..

[18] Scheff S.W., Price D.A., Schmitt F.A., DeKosky S.T., Mufson E.J. (2007). Synaptic alterations in CA1 in mild Alzheimer disease and mild cognitive impairment. Neurology.

[19] Morrison J.H., Baxter M.G. (2012). The ageing cortical synapse: Hallmarks and implications for cognitive decline. Nature Rev. Neurosci..

[20] Zhong L., Cherry T., Bies C.E., Florence M.A., Gerges N.Z. (2009). Neurogranin enhances synaptic strength through its interaction with calmodulin. EMBO J..

[21] Li L., Massimo L., Cole S., Novere N.L., Edelstein S.J. (2020). Neurogranin stimulates Ca2+/calmodulin-dependent kinase II by suppressing calcineurin activity at specific calcium spike frequencies. PLoS Comput. Biol..

[22] Hoffman L., Chandrasekar A., Wand X., Putkey J.A., Waxham M.N. (2014). Neurogranin alters the structure and calmodulin binding properties of calmodulin. J. Biol. Chem..

[23] Bogdanovic N., Davidsson P., Gottfries J., Volkna I., Winblad B., Blennow K. (2002). Regional and cellular distribution of synaptic proteins in the normal human brain. Brain Aging.

[24] Huang K.P., Huang F.L. (2011). Calcium-sensitive translocation of calmodulin and neurogranin between Soma and dendrites of mouse hippocampal CA1 neurons. ACS Chem. Neurosci..

[25] Petersen A., Gerges N.Z. (2015). Neurogranin regulates CaM dynamics at dendritic spines. Sci. Rep..

[26] Davidsson P., Blennow K. (1998). Neurochemical dissection of synaptic pathology in Alzheimer’s disease. Int. Psychogeriatr..

[27] Thorsell A., Bjerke M., Gobom J., Brunhage E., Vanmechelen E., Andreasen N., Hansson O., Minthon L., Zetterberg H., Blennow K. (2010). Neurogranin in cerebrospinal fluid as a marker of synaptic degeneration in Alzheimer’s disease. Brain Res..

[28] Kvartsberg H., Duits F.H., Ingelsson M., Andreasen N., Öhrfelt A., Andersson K., Brinkmalm G., Lannfelt L., Minthon L., Hansson O. (2015). Cerebrospinal fluid levels of the synaptic protein neurogranin correlates with cognitive decline in prodromal Alzheimer’s disease. Alzheimers Dement..

[29] Kester M.I., Teunissen C.E., Crimmins D.L., Herries E.M., Ladenson J.H., Scheltens P., Van Der Flier W.M., Morris J.C., Holtzman D.M., Fagan A.M. (2015). Neurogranin as a cerebrospinal fluid biomarker for synaptic loss in symptomatic Alzheimer disease. JAMA Neurol..

[30] Portelius E., Zetterberg H., Skillbäck T., Törnqvist U., Andreasson U., Trojanowski J.Q., Weiner M.W., Shaw L.M., Mattsson N., Blennow K. (2015). Cerebrospinal fluid neurogranin: Relation to cognition and neurodegeneration in Alzheimer’s disease. Brain.

[31] Mattsson N., Insel P.S., Palmqvist S., Portelius E., Zetterberg H., Weiner M., Blennow K., Hansson O. (2016). Cerebrospinal fluid tau, neurogranin, and neurofilament light in Alzheimer’s disease. EMBO Mol. Med..

[32] Chang J.W., Schumacher E., Coulter P.M., Vinters H.V., Watson J.B. (1997). Dendritic translocation of RC3/neurogranin mRNA in normal aging, Alzheimer disease and fronto-temporal dementia. J. Neuropathol. Exp. Neurol..

[33] George A.J., Gordon L., Beissbarth T., Koukoulas I., Holsinger R.M., Perreau V., Cappai R., Tan S.-S., Masters C.L., Scott H.S. (2010). A serial analysis of gene expression profile of the Alzheimer’s disease Tg2576 mouse model. Neurotox. Res..

[34] D’Alcantara P., Schiffmann S.N., Swillens S. (2003). Bidirectional synaptic plasticity as a consequence of interdependent Ca2+-controlled phosphorylation and dephosphorylation pathways. Eur. J. Neurosci..

[35] Stefan M.I., Edelstein S.J., Le Novere N. (2008). An allosteric model of calmodulin explains differential activation of PP2B and CaMKII. Proc. Natl. Acad. Sci. USA.

[36] Martzen M.R., Slemmon J.R. (1995). The dendritic peptide neurogranin can regulate a calmodulin-dependent target. J. Neurochem..

[37] Penny C.J., Gold M.G. (2018). Mechanisms for localising calcineurin and CaMKII in dendritic spines. Cell Signal..

[38] Takemoto-Kimura S., Suzuki K., Horigane S.-I., Kamijo S., Inoue M., Sakamoto M., Fujii H., Bito H. (2017). Calmodulin kinases: Essential regulators in health and disease. J. Neurochem..

[39] Popugaeva E., Pchitskaya E., Bezprozvanny I. (2017). Dysregulation of neuronal calcium homeostasis in Alzheimer’s disease—A therapeutic opportunity?. Biochem. Biophys. Res. Commun..

[40] Reese L.C., Laezza F., Woltjer R., Taglialatela G. (2001). Dysregulated phosphorylation of (Ca^2+^)/calmodulin-dependent protein kinase II-alpha in the hippocampus of subjects with mild cognitive impairment and Alzheimer’s disease. J. Neurochem..

[41] Ghosh A., Geise K.P. (2015). Calcium/calmodulin-dependent kinase II and Alzheimer’s disease. Mol. Brain.

[42] Taglialatella G., Rastellini C., Cicalese L. (2015). Reduced incidence of dementia in solid organ transplant patients treated with calcineurin inhibitors. J. Alzheimers Dis..

[43] Hong H.S., Hwang J.Y., Son S.M., Kim Y.H., Moon M., Inhee M.J. (2010). FK506 reduces amyloid plaque burden and induces MMP-9 in AbPP/PS1 double transgenic mice. J. Alzheimers Dis..

[44] Rozkalne A., Hyman B.T., Spires-Jones T.L. (2011). Calcineurin inhibition with FK506 ameliorates dendritic spine density deficits in plaque-bearing Alzheimer model mice. Neurobiol. Dis..

[45] Yabuki Y., Matsuo K., Hirano K., Shimoda Y., Moriguchi S., Fukunaga K. (2017). Combined memantine and donepezil treatment improves behavioural and psychological symptoms of dementia-like behaviours in olfactory bulbectomized mice. Pharmacology.

[46] Wang R., Reddy P.H. (2017). Role of glutamate and NMDA receptors in Alzheimer’s disease. J. Alzheimers Dis..

[47] Cline E.N., Bicca M.A., Viola K.L., Klein W.L. (2018). The amyloid-β oligomer hypothesis: Beginning of the third decade. J. Alzheimers Dis..

[48] Hu N.W., Klyubin I., Anwyl R., Rowan M.J. (2009). GluN2B subunit-containing NMDA receptor antagonists prevent Abeta-mediated synaptic plasticity disruption in vivo. Proc. Natl. Acad. Sci. USA.

[49] Malinow R. (2012). New developments on the role of NMDA receptors in Alzheimer’s disease. Curr. Opin. Neurobiol..

[50] Hulme S.R., Jones O.D., Abraham W.C. (2013). Emerging roles of metaplasticity in behaviour and disease. Trends Neurosci..

[51] Opazo P., da Silva S.V., Carta M., Breillat C., Coultrap S.J., Grillo-Bosch D., Sainlos M., Coussen F., Bayer K.U., Mulle C. (2018). CaMKII metaplasticity drives Aβ oligomer-mediated synaptotoxicity. Cell Rep..

[52] Gu Z., Liu W., Yan Z. (2009). Beta-Amyloid impairs AMPA receptor trafficking and function by reducing Ca2+/calmodulin-dependent protein kinase II synaptic distribution. J. Biol. Chem..

[53] Zhao D., Watson J.B., Xie C.W. (2004). Amyloid beta prevents activation of calcium/calmodulin-dependent protein kinase II and AMPA receptor phosphorylation during hippocampal long-term potentiation. J. Neurophysiol..

[54] Ehlers M.D., Zhang S., Bernhardt J.P., Huganir R.L. (1996). Inactivation of NMDA receptors by direct interaction of calmodulin with the NR1 subunit. Cell.

[55] Ataman Z.A., Gakhar L., Sorenson B.R., Hell J.W., Shea M.A. (2007). The NMDA receptor NR1 C1 region bound to calmodulin: Structural insights into functional differences between homologous domains. Structure.

[56] Ehlers M.D., Tingley W.G., Huganir R.L. (1995). Regulated Subcellular Distribution of the NR1 Subunit of the NMDA Receptor. Science.

[57] Zhang S., Ehlers M.D., Bernhardt J.P., Su C.T., Huganir R.L. (1998). Calmodulin mediates calcium-dependent inactivation of N-methylD-aspartate receptors. Neuron.

[58] Iacobucci G.J., Popescu G.K. (2017). Resident calmodulin primes NMDA receptors for Ca^2+^-dependent inactivation. Biophys. J..

[59] Lee J.H., Lee J., Choi K.Y., Hepp R., Lee J.-Y., Lim M.K., Chatani-Hinze M., Roche P.A., Kim D.G., Ahn Y.S. (2008). Calmodulin dynamically regulates the trafficking of the metabotropic glutamate receptor mGluR5. Proc. Nat. Acad. Sci. USA.

[60] Jin D.-Z., Guo M.-L., Xue B., Mao L.-M., Wang J.Q. (2013). Differential regulation of CaMKIIa interactions with mGluR5 and NMDA receptors by Ca^2+^ in neurons. J. Neurochem..

[61] Krucker T., Siggins G.R., McNamara R.K., Lindsley K.A., Dao A., Allison D.W., De Lecea L., Lovenberg T.W., Sutcliffe J.G., Gerendasy D.D. (2002). Targeted disruption of RC3 reveals a calmodulin-based mechanism for regulating metaplasticity in the hippocampus. J. Neurosci..

[62] Zhong L., Gerges N.Z. (2012). Neurogranin targets calmodulin and lowers the threshold for the induction of long-term potentiation. PLoS ONE.

[63] Zhong L., Kaleka K.S., Gerges N.Z. (2011). Neurogranin phosphorylation fine-tunes long-term potentiation. Eur. J. Neurosci..

[64] Berrocal M., Sepulveda M.R., Vazquez-Hernandez M., Mata A.M. (2012). Calmodulin antagonizes amyloid-β peptides-mediated inhibition of brain plasma membrane Ca^2+^-ATPase. Biochim. Biophys. Acta..

[65] Strehler E.E. (2013). Plasma membrane calcium ATPases as novel candidates for therapeutic agent development. J. Pharm. Pharmaceut. Sci..

[66] Corbacho I., Berrocal M., Torok K., Mata A.M., Gutierrez-Merino C. (2017). High affinity binding of amyloid β-peptide to calmodulin: Structural and functional implications. Biochem. Biophys. Res. Commun..

[67] Berrocal M., Corbacho I., Sepulveda M.R., Gutierrez-Merino C., Mata A.M. (2017). Phospholipids and calmodulin modulate the inhibition of PMCA activity by tau. Biochim. Biophys. Acta..

[68] Kushnir A., Wajsberg B., Marks A.R. (2018). Ryanodine receptor dysfunction in human disorders. Biochim. Biophys. Acta.

[69] Chami M., Checler F. (2014). Ryanodine receptors: Dual contribution to Alzheimer disease?. Channels.

[70] Oulès B., Prete D.D., Greco B., Zhang X., Lauritzen I., Sevalle J., Moreno S., Paterlini-Bréchot P., Trebak M., Checler F. (2012). Ryanodine receptors blockade reduces Amyloid-beta load and memory impairments in Tg2576 mouse model of Alzheimer disease. J. Neurosci..

[71] Lanner J.T., Georgiou D.K., Joshi A.D., Hamilton S.L. (2010). Ryanodine receptors: Structure, expression, molecular details, and function in calcium release. Cold Spring Harbor Perspect. Biol..

[72] Rodney G.G., Williams B.Y., Strasburg G.M., Beckingham K., Hamilton S.L. (2000). Regulation of RYR1 activity by Ca2+ and calmodulin. Biochemistry.

[73] Yuchi Z., Kimlicka L., Petegem F.V. (2012). Structural insights into disease mutations of the ryanodine receptor. Genetic Disorders, Chapter 5.

[74] Ferreiro E., Oliviera C.R., Pereira C. (2004). Involvement of endoplasmic reticulum Ca^2+^ release through ryanodine and inositol 1,4,5-triphosphate receptors in the neurotoxic effects induced by the amyloid-beta peptide. J. Neurosci. Res..

[75] Querfurth H.W., Jiang J., Geiger J.D., Selkoe D.J. (1997). Caffeine stimulates amyloid beta peptide release from beta-amyloid precursor protein-tranfected HEK293 cells. J. Neurochem..

[76] McCauley M.D., Wehrens X.H.T. (2011). Ryanodine receptor phosphorylation, calcium/calmodulin-dependent protein kinase II and life-threatening arrhythmias. Trends Cardiovasc. Med..

[77] Berna-Erro A., Jardin I., Salido G.M., Rosado J.A. (2017). Role of STIM2 in cell function and physiopathology. J. Physiol..

[78] Berridge M.J. (1995). Capacitative calcium entry. Biochem. J..

[79] Putney J.W. (1986). A model for receptor-regulated calcium entry. Cell Calcium.

[80] Desai P.N., Zhang X., Wu S., Janoshazi A., Bolimuntha S., Putney J.W., Trebak M. (2015). Multiple types of calcium channels arising from alternative translation initiation of the Orai1 message. Sci. Signal..

[81] Sun S., Zhang H., Liu J., Popugaeva E., Xu N.J., Feske S., White C.L., Bezprozvanny I. (2014). Reduced synaptic STIM2 expression and impaired store-operated calcium entry cause destabilization of mature spines in mutant presenilin mice. Neuron.

[82] Li X., Wu G., Yang Y., Fu S., Liu X., Kang H., Yang X., Su X.-C., Shen Y. (2018). Calmodulin dissociates the SITM1-Orai complex and STIM1 oligomers. Nature commun..

[83] Kwon Y., Hofmann T., Montell C. (2007). Integration of phosphoinositide- and calmodulin-mediated regulation of TRPC6. Mol. Cell..

[84] Lu R., He Q., Wang J. (2017). TRPC channels and Alzheimer’s disease. Adv. Exp. Med. Biol..

[85] Rahman A. (2009). The role of adenosine in Alzheimer’s disease. Curr. Neuropharm..

[86] Arendash G.W., Schleif W., Rezai-Zadeh K., Jackson E.K., Zacharia L.C., Cracchiolo J.R., Shippy D., Tan J. (2006). Caffeine protects Alzheimer’s mice against cognitive impairment and reduce brain β-amyloid production. Neuroscience.

[87] Laurent C., Burnouf S., Ferry B., Batalha V.L., Coelho J.E., Baqi Y., Malik E., Mariciniak E., Parrot S., Van der Jeugd A. (2016). A2A adenosine receptor deletion is protective in a mouse model of Tauopathy. Mol. Psych..

[88] Piirainen H., Hellman M., Tossavainen H., Permi P., Kursula P., Jaakola V.-P. (2015). Human adenosine A2A receptor binds calmodulin with high affinity in a calcium-dependent manner. Biophys. J..

[89] Woods A.S., Marcellino D., Jackson S.N., Franco R., Ferré S., Agnati L.F., Fuxe K. (2008). How calmodulin interacts with the adenosine A(2A) and dopamine D(2) receptors. J. Proteome Res..

[90] Hsiao K., Chapman P., Nilsen S., Eckman C., Harigaya Y., Younkin S., Yang F., Cole G. (1996). Correlative memory deficits, Abeta elevation, and amyloid plaques in transgenic mice. Science.

[91] Dudek N.L., Dai Y., Muma N.A. (2008). Protective effects of interrupting the binding of calmodulin to mutant huntingtin. J. Neuropathol. Exp. Neurol..

[92] Dudek N.L., Dai Y., Muma N.A. (2010). Neuroprotective effects of calmodulin peptide 76-121aa: Disruption of calmodulin binding to mutant huntingtin. Brain Pathol..

[93] Wang R., Yin Y.-X., Mahmood Q., Wang X.-J., Gao Y.-P., Gou G.-J., Ahmed M.M., Kohji F., Du Y.-Z., Han F. (2017). Calmodulin inhibitor ameliorates cognitive dysfunction via inhibiting nitrosative stress and NLRP3 signaling in mice with bilateral carotid artery stenosis. CNS Neurosci. Therap..

[94] Wang X.-J., Gao Y.-P., Lu N.-N., Li W.-S., Xu J.-F., Ying X.-Y., Wu G., Liao M.-H., Tan C., Shao L.-X. (2016). Endogenous polysialic acid based micelles for calmodulin antagonist delivery against vascular dementia. ACS Appl. Mater. Interfaces.

[95] Jung H.H., Kim J.H., Shim J.S., Kwon H.J. (2010). A novel Ca2+/calmodulin antagonist HBC inhibits angiogenesis and down-regulated hypoxia-inducible factor. J. Biol. Chem..

[96] Yuan K., Yong S., Xu F., Zhou T., McDonald J.M., Chen Y. (2015). Calmodulin antagonists promote TRA-8 therapy resistant pancreatic cancer. Oncotarget.

[97] Beuverger P., Ozoux M.-L., Gegis G., Glenat V., Briand V., Philippo M.-C., Daveu C., Tavares G., Roy S., Corbier A. (2020). Reversion of cardiac dysfunction by a novel orally available calcium/calmodulin-dependent protein kinase II inhibitor, RA306, in a genetic model of dilated cardiomyopathy. Cardiovasc. Res..

